# Protocol: an improved method for inducing sporophyte generation in the model moss *Physcomitrium patens* under nitrogen starvation

**DOI:** 10.1186/s13007-023-01077-z

**Published:** 2023-09-26

**Authors:** Emiko Yoro, Shizuka Koshimizu, Takashi Murata, Keiko Sakakibara

**Affiliations:** 1 Department of Life Science, Rikkyo University, 3-34-1, Nishi-Ikebukuro, Toshima-ku, Tokyo, 171-8501 Japan; 2 Division of Evolutionary Biology, National Institute for Basic Biology (NIBB), Okazaki, 444-8585 Japan; 3 Present Address: Bioinformation & DDBJ Center, National Institute of Genetics (NIG), Mishima, 411-8540 Japan; 4 Present Address: Department of Applied Bioscience, Kanagawa Institute of Technology, Atsugi, Kanagawa 243-0292 Japan

**Keywords:** *Physcomitrium patens*, Sporophyte, Nitrogen, Nitrate, Ammonium

## Abstract

**Background:**

Land plants exhibit a haplodiplontic life cycle, whereby multicellular bodies develop in both the haploid and diploid generations. The early-diverging land plants, known as bryophytes, have a haploid-dominant life cycle, in which a short-lived multicellular body in the diploid generation, known as the sporophyte, develops on the maternal haploid gametophyte tissues. The moss *Physcomitrium* (*Physcomitrella*) *patens* has become one of the most powerful model systems in evolutionary plant developmental studies. To induce diploid sporophytes of *P. paten*s, several protocols are implemented. One of the conventional approaches is to grow approximately one-month-old gametophores for another month on Jiffy-7 pellets made from the peat moss that is difficult to fully sterilize. A more efficient method to obtain all tissues throughout the life cycle should accelerate studies of *P. paten*s.

**Results:**

Here, we investigated the effect of nitrogen conditions on the growth and development of *P. patens*. We provide an improved protocol for the sporophyte induction of *P. patens* using a BCD-based solid culture medium without Jiffy-7 pellets, based on the finding that the formation of gametangia and subsequent sporophytes is promoted by nitrogen-free growth conditions. The protocol consists of two steps; first, culture the protonemata and gametophores on nitrogen-rich medium under continuous light at 25 °C, and then transfer the gametophores onto nitrogen-free medium under short-day and at 15 °C for sporophyte induction. The protocol enables to shorten the induction period and reduce the culture space.

**Conclusions:**

Our more efficient and shortened protocol for inducing the formation of sporophytes will contribute to future studies into the fertilization or the diploid sporophyte generation of *P. patens*.

**Supplementary Information:**

The online version contains supplementary material available at 10.1186/s13007-023-01077-z.

## Background

The two main monophyletic clades of land plants, the bryophytes and the vascular plants, diverged more than 400 million years ago [1]. Comparative functional studies of orthologous genes between these two clades have provided important insights into the evolution of plant development, physiology, and metabolism. One of the significant differences between the two groups is the dominant phase of their life cycles; bryophytes have a gametophyte-dominant life cycle, whereas tracheophytes have a sporophyte-dominant life cycle [2, 3], indicating the evolutionary shift from gametophyte dominance to sporophyte dominance. Along with this shift in dominance, it is assumed that some genes controlling gametophyte development are co-opted for sporophyte development, based on the comparative functional studies of orthologous genes, such as *ROOT HAIR DEFECTIVE SIX-LIKE* (*RSL*) and *CLAVATA* (*CLV*) [4–8]. Orthologs of these genes in bryophytes function in the gametophytic tissues, while those in the tracheophyte *Arabidopsis thaliana* are active in the root or shoot, both sporophytic tissues. On the other hand, some genes are specifically expressed in the gametes or sporophytes of both bryophytes and tracheophytes, such as *KNOX* and *BELL* [9–13]. These gene functions are consistently associated with either gamete or sporophyte development, indicating they evolved in the common ancestor before the divergence of the bryophytes and tracheophytes. The transcriptomes of bryophyte sporophytes are quite different from those of their gametophytes, although they are less well characterized due to a research focus on the dominant gametophyte stage [14–16]. Further effective observation and analysis of the sporophyte generations of the bryophytes could therefore provide a better understanding for comparative studies.

In the moss *Physcomitrium patens* (*P. patens*), formerly called *Physcomitrella paten*s [17, 18], sporophytes in the diploid generations develop from the zygote produced following the fertilization of the gametes produced by the gametophytes. It has been previously shown that low temperatures and short-day conditions are critical for the formation of gametes and subsequent sporophytes in *P. patens* [19–21]. In addition to the temperature and light conditions, nutritional status affects the transition to the reproductive phase. Across the green plants, nutritional conditions, especially nitrogen concentration, are presumed to affect the transition to the reproductive phase. *Chlamydomonas reinhardtii*, a unicellular green alga, differentiates into plus or minus gametes in response to nitrogen-depleted conditions [22, 23]. The flowering times of the angiosperms are also affected by their nitrogen status [24, 25]. This inspired research interest into the effects of nitrogen depletion on the efficient transition into the reproductive phase and subsequent sporophyte development in *P. patens*.

Conventionally, sexual reproduction in *P. patens* has been conducted with several protocols by using Jiffy‐7 peat pellets (Jiffy Products International AS, Kristansand, Norway) [9, 15, 26, 27], Knop’s-based medium [20, 28], or BCD-based medium [21, 29–31]. These conventional protocols remain several problems. Firstly, in the original method by Hohe et al., it takes 7 weeks before gametangia are formed on the isolated gametophores grown on the Knop’s-based medium. Compared with to the original method, an alternative method by Jiffy‐7 pellets which enable to shorten the period for inducing gametangia: from 7 to 3 weeks. However, the Jiffy‐7 pellets contains unknown contaminants that were always detected when we transferred the plants grown on the pellets into solid agar medium, even after sterilization for 40 min at 120 °C (Additional file 1: Fig. S1). The second important point is the period of pre-culture at 25 °C to grow gametophores before transferring to gametangia-inductive conditions. It takes 4–6 weeks in all kinds of conventional methods [9, 21, 26, 27, 30–32]. The third points are the culture spaces. Both a plastic plant box (75 × 75 × 100 mm; AS ONE, Osaka, Japan) used for Jiffy and 220 ml bulbous Weck jars (Weck, Wehr-Öflingen, Germany) used for solidified media require larger space in incubators. These methods have still room for improvement, including the manipulation, the reduction of culture time and culture space, and the exclusion of unknown contaminants. Lastly, another problem is a low rate of sporophyte formation in the Gransden2004 strain [33, 34]. Although the Reute accession that exhibits the higher rate has used for fertilization assays as an alternative way [29, 30, 33] the techniques to apply across multiple accessions containing Gransden-based strain would be still valuable for research communities.

In this study, we focus on the importance of low nitrogen in gametangia and subsequent sporophyte formation, using the Cove-NIBB strain that shows a higher efficiency of sporophyte formation and derived from a common single spore with the world-wide Gransden2004 strain [35]. Previously, several reports have indicated an importance of the reduced nitrogen in reproductive phase [36–38]; however, the actual effects and the optimal conditions of nitrogen for sporophyte formation have not been clarified. Here, we investigate the growth effects of nitrogen on both the haploid and diploid generations. Based on the results, we optimize the conditions and provide an improved protocol enabling us to obtain sporophytes more quickly and easily. The method should be helpful for studies in this model species.

## Results and discussion

### Growth effects of different nitrogen sources on protonemata and gametophores

**Fig. 1:**
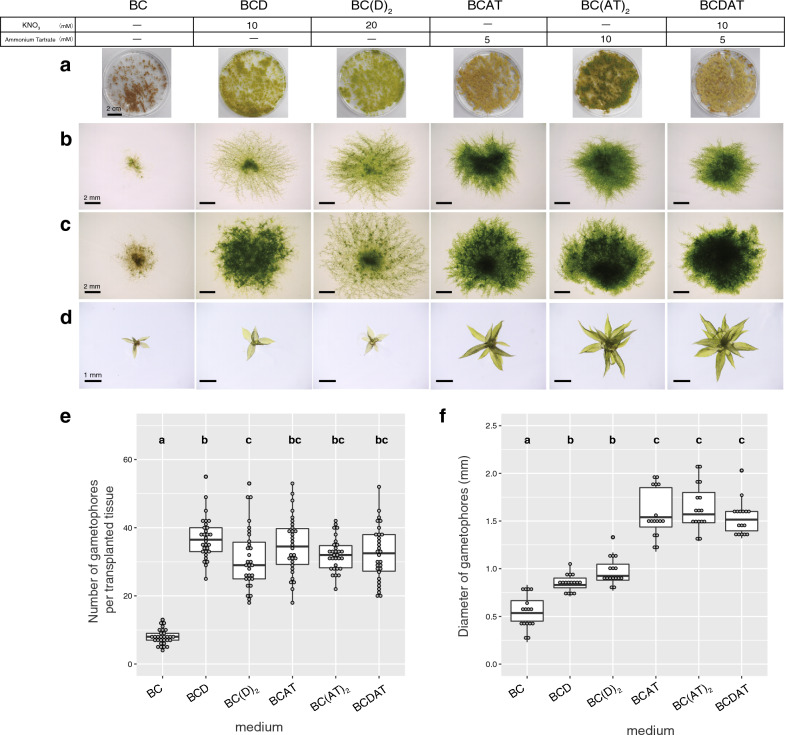
Effects of the nitrogen sources and their concentrations on gametophyte growth. **a** Overview of protonemata at 30 days after homogenization. **b**, **c** Magnified tissues with gametophores at 10 days (**b**) or 18 days (**c**) after transplantation. **d** Top view of gametophores isolated from the 18-days-old tissues grown on the respective media. **e** Boxplot of the number of gametophores per single tissue at 18 days after transplantation (n = 30). **f** Boxplot of the diameter of the top view of the gametophores isolated from the tissues, as shown in (**d**) at 18 days after transplantation (n = 16). Different lowercase letters represent statistically significant differences (*P* < 0.05; Tukey’s HSD)

The model moss *P. patens* has been cultured on inorganic salt media, or BCD-based [35, 39] or KNOP-based media [20, 40]. To examine the effects of nitrogen sources on the growth of *P. patens*, we prepared a series of BCD-based media with or without stock solution D (containing KNO_3_) and (di)ammonium tartrate (AT), termed BC, BCDAT, BCD, BC(D)_2_, BC(D)_5_, BCAT, BC(AT)_2_, and BC(AT)_5_. The nitrogen compositions of the culture media are summarized in the top part of Fig. 1. In addition, the detailed compositions of the series of media were shown in Additional file 1: Tables S1, S2. We first examined the growth and developmental effects of nitrogen on haploid gametophyte tissues. The vegetative haploid gametophyte tissues of *P. patens* are filamentous protonemata and leafy gametophores, with the protonemata characterized into two main types: chloronemata, containing large round green chloroplasts, and chloroplast-poor brownish caulonemata [41, 42]. The preserved protonemata were freshly homogenized and cultured on a series of BCD-based media. After a 30 day culture, the protonemal tissues on BC, BCD, BCAT, and BCDAT turned brownish, but the tissues on the media containing additional nitrogen sources (BC(D)_2_ and BC(AT)_2_) remained green in color (Fig. 1a). Those green tissues might be related to the previous reports that the presence of additional ammonium delays the chloronema‐to‐caulonema transition [37, 43, 44]; alternatively, be explained by the amount of available nitrogen.

Next, to investigate whether the nitrogen influences on the gametophore formation, a small piece of freshly prepared 5-days-old protonema grown on the standard BCDAT medium was transplanted onto a series of 30 ml nitrogen media. At 10 days after transplantation, one of them is shown (Fig. 1b). The density of protonemal tissue was lower on BCD or BC(D)_2_ than that on ammonium supplemented medium, consistent with the growth-promotion effect of ammonium [45, 46]. At 18 days after transplantation, the number of gametophores on the nitrogen-free BC medium was markedly reduced, as expected, whereas the numbers present on the other media were not significantly changed (Fig. 1c, e). On nitrogen-free BC media, protonema elongation and gametophore development are significantly reduced by the nutrient deficiency, but rhizoid differentiation is accelerated (Fig. 1b, c). At least in our condition, the number of gametophores was not significantly changed between BCD (0 mM AT) and BCDAT (5 mM AT) (Fig. 1f); however, the lower concentration of ammonium (less than 5 mM AT) could increase the numbers [47].

Furthermore, to evaluate the growth of gametophores in different nitrogen concentration, we chose one mass of tissue containing protonema and gametophores in each condition and randomly picked up gametophores (n = 16) by forceps and examined the growth of them by their diameter shown from the top (Fig. 1e). Importantly, the diameter was larger on the AT-containing media (BCDAT, BCAT, and BC(AT)_2_) than on the AT-free media (BC, BCD, and BC(D)_2_) (Fig. 1e–g), indicating that ammonium promotes the growth of gametophores. These positive effects of AT on gametophore growth are consistent with previous findings [45, 46]. A significant increase in size of gametophores on BCDAT provides information of improvement of culture condition, such as reduction in incubation periods and efficient gametangia induction, thereby we concluded that BCDAT is more suitable than BCD for gametophore induction. According to the previous description, larger gametophores tended to induce gametangia faster [48].

### Nitrogen-free growth condition accelerates sporophyte development

**Fig. 2:**
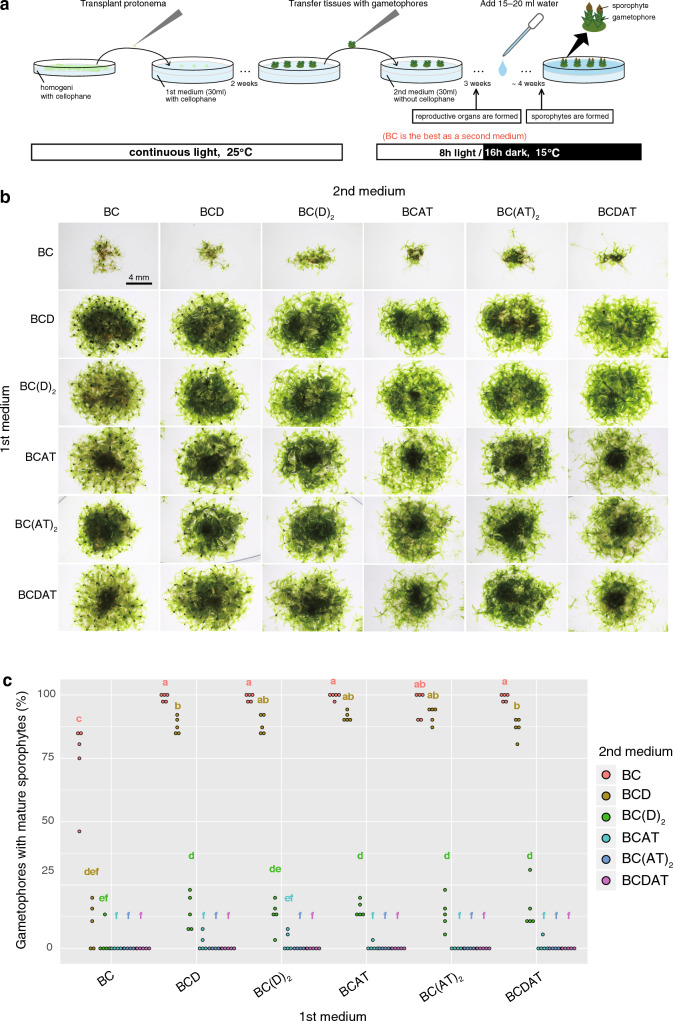
Effects of nitrogen sources and their concentrations on sporophyte formation. **a** Schematic visual explanations of the method used for sporophyte induction in this study. **b** Magnified images of tissues containing gametophores with or without sporophytes at 8 weeks after transfer onto the second medium. Scales are the same between panels. **c** Dot plot of the formation rate of brownish mature sporophytes per tissue (the number of gametophores with mature sporophytes/total number of gametophores). The results of five tissues in each condition are shown. Different lowercase letters represent statistically significant differences (*P* < 0.05; Tukey’s HSD)

To evaluate the effect of nitrogen on the formation of reproductive organs and diploid sporophyte generation, gametophores grown on the series of media with varying nitrogen contents were further transplanted onto different media (hereafter referred to as the first medium for gametophore induction and the second medium for sporophyte induction, respectively) (Fig. 2a). To prevent protonemal tissues from invading the first medium, we planted a small piece of protonema on the medium fully overlaid with cellophane. Thanks to the cellophane, the obtained tissues indirectly connected to the medium were flat-bottomed, allowing the easy transfer onto the second medium. After transplantation onto the second medium, the plates are transferred into 15 °C with eight hours of light and 16 h of darkness. After 8 weeks of incubation on the second medium, brown-colored mature sporophytes were observed on the AT-free media (BC, BCD, or BC(D)_2_), regardless of the first medium used (Fig. 2b). By contrast, sporophytes were very rarely observed on the gametophores grown on the AT-containing second media. The few gametophores grown on BC as a first medium also formed mature sporophytes when these were transferred onto an AT-free second medium. The percentage of gametophores with mature sporophytes were calculated, and the data from five masses of tissues in each series of media are shown (Fig. 2c). The rate was markedly decreased in BC(D)_2_ than in BC or BCD, suggesting that sporophyte formation is strongly inhibited by additional KNO_3_. The statistical analysis indicates that both the ratio of BC and BCD are almost the same level.

**Fig. 3:**
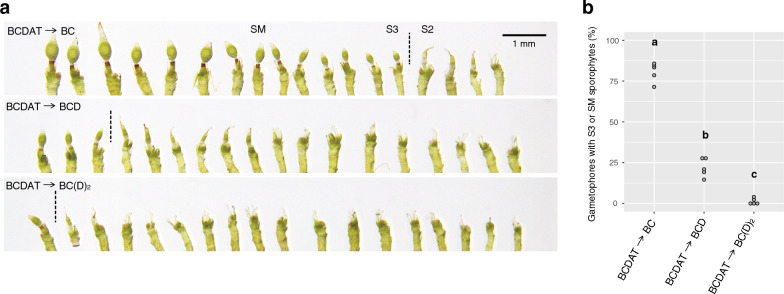
Faster sporophyte development on nitrogen-free medium. **a** Sporophyte developmental orders of 18 gametophores from a single protonemal tissue after 6 weeks of growth on the second medium. The leaves are removed to expose the gametangia or sporophytes. The media used are indicated on each panel (from the first medium to the second medium). Dashed lines indicate the border between the sporophyte developmental stages S2 and S3. **b** Dot plot of the ratio of intermediate and mature sporophytes (stages S3 or S3M) to the total number of gametophores (data from five tissues). Different lowercase letters represent statistically significant differences (*P* < 0.05; Tukey’s HSD)

To further explore the effects of nitrogen-free conditions for the acceleration of sporophyte development, we randomly picked 18 gametophores from a single mass of tissue at an earlier stage of sporophyte development (after 6 weeks of incubation on the second media) and aligned them in developmental order (Fig. 3a). The mass of tissue on BC as a second medium harbored more sporophytes at a later stage than those grown on BCD or BC(D)_2_, even though they were grown on the same first medium (BCDAT). This result shows that, rather than the final percentage of sporophyte formation, the development of the reproductive organs or sporophytes was accelerated on nitrogen-free conditions. To determine the delay in the development of sporophytes caused by the additional nitrates, we measured the ratio of the well-developed sporophytes without calyptra (stages S3 and SM [15]) to the total number of gametophores. The values were remarkably increased when BC was used as the second medium (Fig. 3b).

### Reproductive cells developed in the media with varying levels of nitrogen

**Fig. 4:**
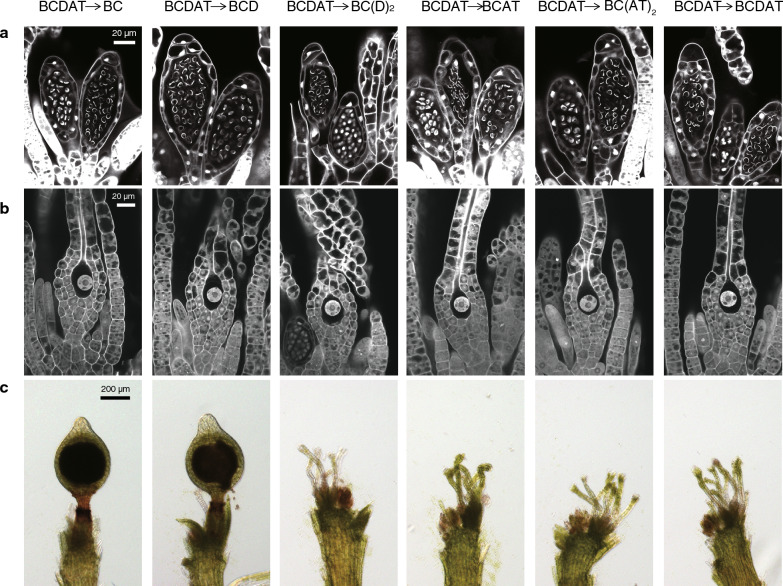
Reproductive cell differentiation of gametophores grown on all nitrogen conditions. **a** Mature antheridia containing differentiated sperms at 2−3 weeks after transfer onto the second medium. **b** Mature archegonia containing an egg at 2−3 weeks after transfer onto the second medium. **c** Stereoscopic images of the gametophore apexes at 8 weeks after transfer onto the second medium. The used media (from the first medium to the second medium) are indicated on the top of each panel

To determine whether the nitrogen contents or conditions affect gametangia development or reproductive cell differentiation, we observed the antheridia and archegonia in a series of different second media. In *P. patens*, gametangia stem cells give rise to the antheridia and later the archegonia [49] in well-described developmental processes [50]. Two to 3 weeks after transfer onto the second medium, no obvious defects in the egg morphology or sperm nucleus condensation were observed between the plants grown on any of the second media (Fig. 4a, b), despite a lack of gametophores with the sporophytes on the AT-containing medium (Figs. 2c, 4c). These results indicate that the presence of AT affects fertilization rather than gametogenesis. On the other hand, the neck cells of the non-fertile archegonia on the AT-containing media are green, indicating a delayed degradation of chloroplasts, as previously observed in the *Ppshi2* (*short internode2/stylish2*) mutant lines [50]. This suggests a potential negative effect of AT during the maturation of the egg cell or archegonium. Further experiments demonstrating the motility and viability of the sperm, or the fertilization ability of the egg are necessary to elucidate the effects of nitrogen contents or conditions on sperm and egg differentiation. Experiments using previously isolated mutants defective in fertilization [26, 27, 51] may help to answer these questions. Low nitrogen promotes gametangia formation in several bryophytes, including *Riccia* liverworts [36]; however, whether these effects are common in the bryophytes is still an intriguing question.

### Protocol: easier and faster induction of *P. patens* sporophytes

- 1. Prepare freshly homogenized five-day-old protonemal tissue.
- 2. Transplant a piece of protonemal tissue (2−5 mm diameter) onto a cellophane-overlaid 30 ml BCDAT agar medium in a φ 90 × 20 mm plastic Petri dish (the first medium). Seal 3/4 of the Petri dish with Parafilm M and cover the remaining gap with 3 M Micropore Tape. Tissues on cellophane can be moved, so take care not to mix the tissues when planting multiple different strains on a single plate. The maximum number of pieces of tissues are 25 per plate.
- 3. Incubate plates for 2 weeks at 25 °C under continuous light. To induce approximately 20−50 gametophores in each mass of tissues, it takes at least 14 days. The incubation periods can be extended for several weeks, depending on the tissue conditions.
- 4. Transfer the tissues containing gametophores onto 30 ml of nitrogen-free BC agar medium in a φ 90 × 20 mm plastic Petri dish (the second medium) without cellophane. Seal 3/4 of the Petri dish with Parafilm M and cover the remaining gap with 3 M Micropore Tape.
- 5. Incubate plates at 15 °C under a photoperiod with 8 h of light and 16 h of darkness. Antheridia containing mature sperms develop on the 10th day, while distinguishable archegonia (stage 5) start to develop on the 14th day.
- 6. Three weeks after transferring onto the second medium, add 15–20 ml sterilized water to each plate to promote fertilization. Seal the Petri dish with Parafilm M to prevent desiccation and contamination. The sporophytes will be formed even if you do not add water (Fig. 2a).

### Comments about our improved protocol

At first, as mentioned previously, the Gransden2004 strain displayed a low rate of sporophyte formation [33, 34]. We therefore used the Cove-NIBB (also known as David-NIBB) [35]. According to the previous reports using Jiffy-7 pellets, the rate of sporophyte formation by wild type of the Cove-NIBB is around 60−80% [26, 27, 52, 53]. In our improved method using a BC solid medium, we succeeded in obtaining a higher rate of sporophyte formation (approximately 100%) (Fig. 2c). The high rate is comparable with that observed in Reute accession [30, 31, 33].

Other than the high rate of sporophyte induction compared with multiple conventional methods, our protocol provides the following benefits: (1) faster sporophyte induction, (2) less incubation space required, (3) less protonema tissue required for gametophyte induction, and (4) acquisition of completely sterilized gametangia and sporophytes. Compared with all conventional procedures, our improved protocol using nitrogen-rich BCDAT medium shortens the period of pre-culture for growing gametophores at 25 °C, from 4 to 6 weeks in the conventional methods to 2 weeks in our improved method. This contributes to the shortening of the total period required for obtaining sporophytes; 2–3 weeks faster than that of the conventional methods. In terms of space-saving, Petri dishes are better than the plastic box used for Jiffy-7 pellets or Weck jars used for some agar-based methods. The smaller Petri dishes (φ 55 × 12 mm) are also available, enabling the incubation of sporophytes from many strains at the same time for uses such as large-scale screening. For saving protonemal tissues for the pre-culture, a small piece of protonemata (2–5 mm diameter) is sufficient for formation of a single mass of tissue inducing 20–50 gametophores. This manipulation that is almost the same as in the previous procedures established by Thelander et al. [32]. Finally, the completely sterilized sporophytes obtained using this improved protocol allow for flexibility in experimental design. Completely sterilized spores are very useful not only for self-fertilization and long-term preservation but also for out-crossing between different strains or accessions. As previously shown in several studies of the Reute accession, using our protocol the out-crossing with the world-wide Gransden2004 should be possible, since the low fertility of the Gransden2004 is caused by male side [34]. It will be of interest to examine whether the nitrogen-free conditions recover the male infertility of the Gransden2004.

## Conclusions

The growth of the leafy gametophores of *P. patens* was promoted on nitrogen-rich AT-containing media. Conversely, more sporophytes were formed on the gametophores transferred onto nitrogen-free media. The number of sporophytes was dramatically reduced on the gametophores grown on AT-containing media. Unlike AT, nitrate did not completely inhibit sporophyte formation, but higher concentrations did delay their development. We provide an improved protocol for the induction of sporophytes in *P. patens*: gametophores are pre-grown on BCDAT then transferred to a nitrogen-free BC medium while simultaneously transitioning to low-temperature and short-day conditions. Compared with the previous methods, this protocol will accelerate further studies of *P. patens*, particularly those with a focus on reproductive organs and sporophyte tissues.

## Methods

### Plant materials and growth conditions

The *P. patens* (Hedw.) Bruch et Schimp ‘Cove-NIBB’, ‘David-NIBB’ or Okazaki 1998 strain [26, 35, 39, 54] was used in this study. A variety of media based on the BCDAT medium were assessed. Their nitrogen contents were modified by the addition or exclusion of stock solution D (containing KNO_3_) and (di)ammonium tartrate, as summarized in Additional file 1: Table S2. A 30 ml aliquot of medium was used in each plastic Petri dish (φ 90 × 20 mm; SHI-ATEX, Saijo, Japan). Protonemata and gametophores were grown on the first medium at 25 °C under continuous light. The cultured protonemal tissue with gametophores were then transferred onto the second medium and cultured at 15 °C under 8 h of light and 16 h of darkness to induce gametangia and sporophyte formation.

### Phenotypic observation

The images of Petri dishes and protonemal tissues including gametophores were photographed using a digital camera (EOS6D; Canon, Tokyo, Japan), a microscope over-eyepiece camera, or tereomicroscopes (MZ10F; Leica Microsystems, Wetzlar, Germany) fitted with a DFC450 C Digital Camera. The magnified images of gametangia and sporophytes were observed by inverted microscope (Axio Observer D1, Zeiss).

### Confocal microscopic observation

The reproductive organs were observed by the modified protocol of the previous report [51]. The tissues were fixed overnight in a solution of 4% (v/v) glutaraldehyde and 1 μg per ml DAPI (DOJINDO, Kumamoto, Japan) in 12.5 mM sodium phosphate (pH 7.0) at 4 °C. The fixed materials were then dehydrated in a graded ethanol series and cleared in a 2:1 mixture of benzyl benzoate and benzyl alcohol. The tissues were observed using a laser-scanning confocal microscope (LSM710; Carl Zeiss, Jena, Germany) with a 63 × oil immersion lens. DAPI fluorescence was detected under excitation between 420 and 480 nm with a 405 nm UV laser.

### Graphics and statistical analysis

Box plot and dot plot analyses were performed in R studio (R 4.2.1) using package ggplot2 or basic R plotting commands. In the boxplots, the upper and lower “hinges” correspond to the first and third quartiles. One-way ANOVA and post-hoc Tukey’s HSD has been done by multcompLetters4 function from the multcompView package. Dunnet’s test was done by basic R script and letters were added manually. The graphs were further modified using Adobe illustrator 2023.

### Supplementary Information

**Additional file 1: Fig S1.** Contaminants from Jiffy-7 pellets. (a-c) Images of a part of Jiffy‐7 (a), a gametophore cultured on Jiffy‐7 (b), and protonema before transfer to Jiffy-7 (c) on LB medium. Growth of unknown bacteria was observed in a part of Jiffy‐7 (a) and a gametophore cultured on Jiffy‐7 (b), but not in protonema before transfer to Jiffy-7 (c). (d) An image of scanning electron microscope (SEM) of a leaf of gametophore cultured on Jiffy‐7. White arrows show bacterial clump (blackish one). **Table S1.** Stock solutions used in this study. **Table S2.** BCD-based media used in this study.

## Data Availability

All data generated or analyzed during this study are included in this article. This work was performed with the *P. patens* Gransden Cove-NIBB (or David-NIBB) strain. Although both widely used Gransden2004 and Cove-NIBB share a common single spore ancestor, the frequency of sporophyte development is higher in Cove-NIBB and lower in Gransden2004 (http://www.nibb.ac.jp/evodevo/PHYSCOmanual/1.htm, last visited August 2023).

## Abbreviations

AT: (Di)ammonium tartrate
KNO_3_: Potassium nitrate
DAPI: 4’,6-Diamidine-2’-phenylindole dihydrochloride

## References

[1] Puttick MN, Morris JL, Williams TA, Cox CJ, Edwards D, Kenrick P (2018). The interrelationships of land plants and the nature of the ancestral embryophyte. Curr Biol.

[2] Bowman JL, Sakakibara K, Furumizu C, Dierschke T (2016). Evolution in the cycles of life. Annu Rev Genet.

[3] Kenrick P, Crane PR (1997). The origin and early evolution of plants on land. Nature.

[4] Menand B, Yi K, Jouannic S, Hoffmann L, Ryan E, Linstead P (2007). An ancient mechanism controls the development of cells with a rooting function in land plants. Science.

[5] Honkanen S, Thamm A, Arteaga-Vazquez MA, Dolan L (2018). Negative regulation of conserved RSL class I bHLH transcription factors evolved independently among land plants. Elife.

[6] Pires ND, Yi K, Breuninger H, Catarino B, Menand B, Dolan L (2013). Recruitment and remodeling of an ancient gene regulatory network during land plant evolution. Proc Natl Acad Sci USA.

[7] Moody LA, Kelly S, Rabbinowitsch E, Langdale JA (2018). Genetic regulation of the 2D to 3D growth transition in the moss *Physcomitrella patens*. Curr Biol.

[8] Hirakawa Y, Fujimoto T, Ishida S, Uchida N, Sawa S, Kiyosue T (2020). Induction of multichotomous branching by CLAVATA peptide in *Marchantia polymorpha*. Curr Biol.

[9] Sakakibara K, Nishiyama T, Deguchi H, Hasebe M (2008). Class 1 KNOX genes are not involved in shoot development in the moss *Physcomitrella patens* but do function in sporophyte development. Evol Dev.

[10] Sakakibara K, Ando S, Yip HK, Tamada Y, Hiwatashi Y, Murata T (2013). KNOX2 genes regulate the haploid-to-diploid morphological transition in land plants. Science.

[11] Horst NA, Katz A, Pereman I, Decker EL, Ohad N, Reski R (2016). A single homeobox gene triggers phase transition, embryogenesis and asexual reproduction. Nat Plants.

[12] Hisanaga T, Fujimoto S, Cui Y, Sato K, Sano R, Yamaoka S (2021). Deep evolutionary origin of gamete-directed zygote activation by KNOX/BELL transcription factors in green plants. Elife.

[13] Dierschke T, Flores-Sandoval E, Rast-Somssich MI, Althoff F, Zachgo S, Bowman JL (2021). Gamete expression of tale class hd genes activates the diploid sporophyte program in *Marchantia polymorpha*. Elife.

[14] Frank MH, Scanlon MJ (2015). Transcriptomic evidence for the evolution of shoot meristem function in sporophyte-dominant land plants through concerted selection of ancestral gametophytic and sporophytic genetic programs. Mol Biol Evol.

[15] Ortiz-Ramírez C, Hernandez-Coronado M, Thamm A, Catarino B, Wang M, Dolan L (2016). A transcriptome atlas of *Physcomitrella patens* provides insights into the evolution and development of land plants. Mol Plant.

[16] Flores-Sandoval E, Romani F, Bowman JL (2018). Co-expression and transcriptome analysis of *Marchantia polymorpha* transcription factors supports class C ARFs as independent actors of an ancient auxin regulatory module. Front Plant Sci.

[17] Rensing SA, Lang D, Zimmer AD, Terry A, Salamov A, Shapiro H (2008). The physcomitrella genome reveals evolutionary insights into the conquest of land by plants. Science.

[18] Rensing SA, Goffinet B, Meyberg R, Wu SZ, Bezanilla M (2020). The moss *Physcomitrium* (*Physcomitrella*) *patens*: a model organism for non-seed plants. Plant Cell.

[19] Engel PP (1968). The induction of biochemical and morphological mutants in the moss *Physcomitrella patens*. Am J Bot.

[20] Hohe A, Rensing SA, Mildner M, Lang D, Reski R (2002). Day length and temperature strongly influence sexual reproduction and expression of a novel MADS-box gene in the moss *Physcomitrella patens*. Plant Biol.

[21] Cove DJ, Perroud P-F, Charron AJ, McDaniel SF, Khandelwal A, Quatrano RS (2009). Culturing the moss *Physcomitrella patens*. Cold Spring Harb Protoc.

[22] Sager R, Granick S (1954). Nutritional control of sexuality in *Chlamydomonas reinhardi*. J Gen Physiol.

[23] Park JJ, Wang H, Gargouri M, Deshpande RR, Skepper JN, Holguin FO (2015). The response of *Chlamydomonas reinhardtii* to nitrogen deprivation: a systems biology analysis. Plant J.

[24] Lin YL, Tsay YF (2017). Influence of differing nitrate and nitrogen availability on flowering control in *Arabidopsis*. J Exp Bot.

[25] Sanagi M, Aoyama S, Kubo A, Lu Y, Sato Y, Ito S (2021). Low nitrogen conditions accelerate flowering by modulating the phosphorylation state of FLOWERING BHLH 4 in *Arabidopsis*. Proc Natl Acad Sci USA.

[26] Koshimizu S, Kofuji R, Sasaki-Sekimoto Y, Kikkawa M, Shimojima M, Ohta H (2018). *Physcomitrella* MADS-box genes regulate water supply and sperm movement for fertilization. Nat Plants.

[27] Ortiz-Ramírez C, Michard E, Simon AA, Damineli DSC, Hernández-Coronado M, Becker JD (2017). GLUTAMATE RECEPTOR-LIKE channels are essential for chemotaxis and reproduction in mosses. Nature.

[28] Perroud PF, Meyberg R, Rensing SA (2019). *Physcomitrella patens* Reute mCherry as a tool for efficient crossing within and between ecotypes. Plant Biol.

[29] Thelander M, Landberg K, Sundberg E (2018). Auxin-mediated developmental control in the moss *Physcomitrella patens*. J Exp Bot.

[30] Sanchez-Vera V, Landberg K, Lopez-Obando M, Thelander M, Lagercrantz U, Muñoz-Viana R (2022). The *Physcomitrium patens* egg cell expresses several distinct epigenetic components and utilizes homologues of BONOBO genes for cell specification. New Phytol.

[31] Landberg K, Lopez-Obando M, Sanchez Vera V, Sundberg E, Thelander M (2022). MS1/MMD1 homologues in the moss *Physcomitrium patens* are required for male and female gametogenesis. New Phytol.

[32] Thelander M, Landberg K, Sundberg E (2019). Minimal auxin sensing levels in vegetative moss stem cells revealed by a ratiometric reporter. New Phytol.

[33] Hiss M, Meyberg R, Westermann J, Haas FB, Schneider L, Schallenberg-Rüdinger M (2017). Sexual reproduction, sporophyte development and molecular variation in the model moss *Physcomitrella patens*: introducing the ecotype Reute. Plant J.

[34] Meyberg R, Perroud PF, Haas FB, Schneider L, Heimerl T, Renzaglia KS (2020). Characterisation of evolutionarily conserved key players affecting eukaryotic flagellar motility and fertility using a moss model. New Phytol.

[35] Ashton NW, Raju MVS (2000). The distribution of gametangia on gametophores of *Physcomitrella* (*Aphanoregma*) *patens* in culture. J Bryol.

[36] Chopra RN, Bhatla SC (1983). Regulation of gametangial formation in bryophytes. Bot Rev.

[37] Mohanasundaram B, Pandey S (2022). Effect of environmental signals on growth and development in mosses. J Exp Bot.

[38] Perroud PF, Cove DJ, Quatrano RS, Mcdaniel SF (2011). An experimental method to facilitate the identification of hybrid sporophytes in the moss *Physcomitrella patens* using fluorescent tagged lines. New Phytol.

[39] Nishiyama T, Hiwatashi Y, Sakakibara K, Kato M, Hasebe M (2000). Tagged mutagenesis and gene-trap in the moss, *Physcomitrella patens* by shuttle mutagenesis. DNA Res.

[40] Reski R, Abel WO (1985). Induction of budding on chloronemata and caulonemata of the moss, *Physcomitrella patens*, using isopentenyladenine. Planta.

[41] Mattias T, Tina O, Hans R (2005). Effect of the energy supply on filamentous growth and development in *Physcomitrella patens*. J Exp Bot.

[42] Cove D, Bezanilla M, Harries P, Quatrano R (2006). Mosses as model systems for the study of metabolism and development. Annu Rev Plant Biol.

[43] Jenkins GI, Cove DJ (1983). Light requirements for regeneration of protoplasts of the moss *Physcomitrella patens*. Planta.

[44] Perroud PF, Haas FB, Hiss M, Ullrich KK, Alboresi A, Amirebrahimi M (2018). The *Physcomitrella patens* gene atlas project: large-scale RNA-seq based expression data. Plant J.

[45] Sung HC, Addo-Quaye C, Coruh C, Arif MA, Ma Z, Frank W (2008). Physcomitrella patens DCL3 is required for 22–24 nt siRNA accumulation, suppression of retrotransposon-derived transcripts, and normal development. PLoS Genet.

[46] Martens M, Horres R, Wendeler E, Reiss B (2020). The importance of ATM and ATR in *Physcomitrella patens* DNA damage repair, development, and gene targeting. Genes.

[47] Plavskin Y, Nagashima A, Perroud PF, Hasebe M, Quatrano RS, Atwal GS (2016). Ancient trans-acting siRNAs confer robustness and sensitivity onto the auxin response. Dev Cell.

[48] Horst NA, Reski R (2017). Microscopy of *Physcomitrella patens* sperm cells. Plant Methods.

[49] Kofuji R, Yagita Y, Murata T, Hasebe M (2018). Antheridial development in the moss *Physcomitrella patens*: implications for understanding stem cells in mosses. Phil Trans Royal Soc B.

[50] Landberg K, Pederson ERA, Viaene T, Bozorg B, Friml J, Jönsson H (2013). The moss *Physcomitrella patens* reproductive organ development is highly organized, affected by the two SHI/STY genes and by the level of active auxin in the SHI/STY expression domain. Plant Physiol.

[51] Tanahashi T, Sumikawa N, Kato M, Hasebe M (2005). Diversification of gene function: homologs of the floral regulator FLO/LFY control the first zygotic cell division in the moss *Physcomitrella patens*. Development.

[52] Sakakibara K, Reisewitz P, Aoyama T, Friedrich T, Ando S, Sato Y (2014). WOX13-like genes are required for reprogramming of leaf and protoplast cells into stem cells in the moss *Physcomitrella patens*. Development.

[53] Hashida Y, Takechi K, Abiru T, Yabe N, Nagase H, Hattori K (2020). Two *ANGUSTIFOLIA* genes regulate gametophore and sporophyte development in *Physcomitrella patens*. Plant J.

[54] Haas FB, Fernandez-Pozo N, Meyberg R, Perroud PF, Göttig M, Stingl N, Saint-Marcoux D, Langdale JA, Rensing SA (2020) Single Nucleotide Polymorphism Charting of *P. patens* Reveals Accumulation of Somatic Mutations During in vitro Culture on the Scale of Natural Variation by Selfing. Front Plant Sci. 11:813. 10.3389/fpls.2020.00813. 10.3389/fpls.2020.00813 PMC7358436 32733496

