# Supplementary material for: Protocol: an improved method for inducing sporophyte generation in the model moss Physcomitrium patens under nitrogen starvation
Source: Plant Methods. 2023 Sep 26;19:100. doi: 10.1186/s13007-023-01077-z (PMC10521525; doi:10.1186/s13007-023-01077-z)
Supplement: Supplementary file 1 — Additional file 1: Fig S1. Contaminants from Jiffy-7 pellets. (a-c) Images of a part of Jiffy‐7 (a), a gametophore cultured on Jiffy‐7 (b), and protonema before transfer to Jiffy-7 (c) on LB medium. Growth of unknown bacteria was observed in a part of Jiffy‐7 (a) and a gametophore cultured on Jiffy‐7 (b), but not in protonema before transfer to Jiffy-7 (c). (d) An image of scanning electron microscope (SEM) of a leaf of gametophore cultured on Jiffy‐7. White arrows show bacterial clump (blackish one). Table S1. Stock solutions used in this study. Table S2. BCD-based media used in this study. [file 13007_2023_1077_MOESM1_ESM.zip › Additional file 1/FigS1-f2.pdf]

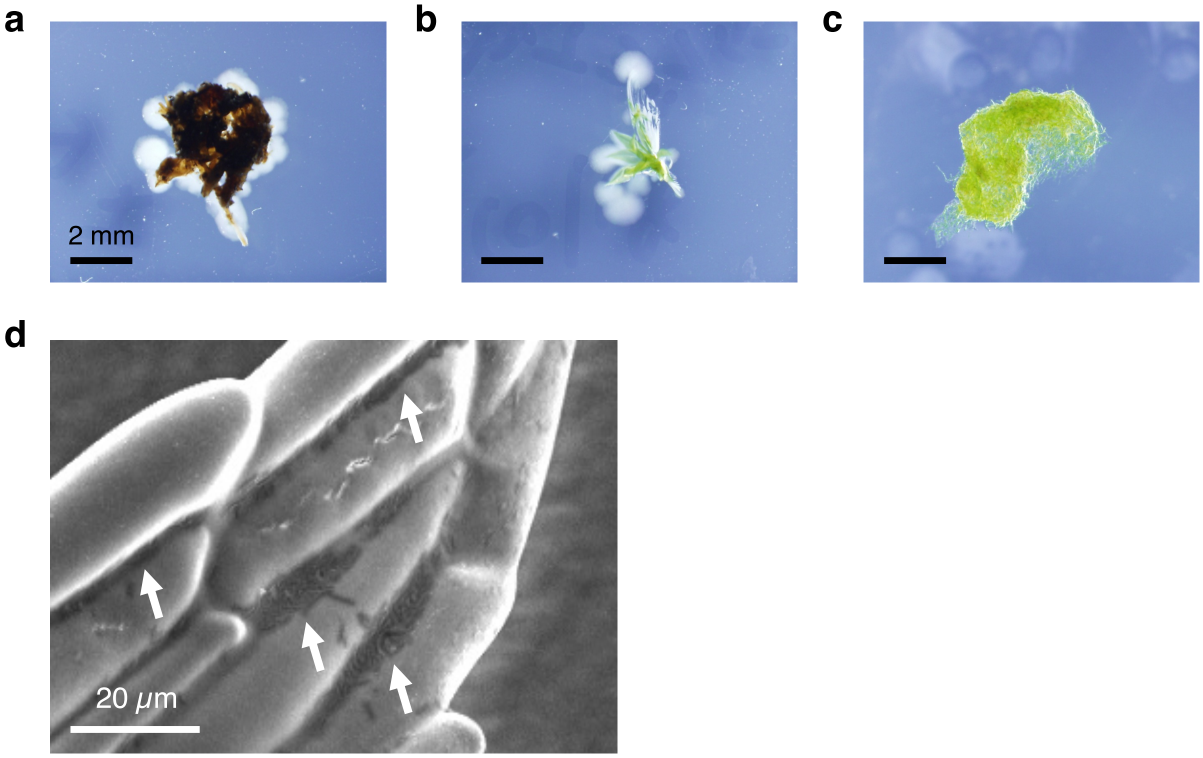

Supplementary Fig. S1 Contaminants from Jiffy-7 pellets.
(a-c) Images of a part of Jiffy-7 (a), a gametophore cultured on Jiffy-7 (b), and protonema before transfer to Jiffy-7 (c) on LB medium. Growth of unknown bacteria was observed in a part of Jiffy-7 (a) and a gametophore cultured on Jiffy-7 (b), but not in protonema before transfer to Jiffy-7 (c). (d) An image of scanning electron microscope (SEM) of a leaf of gametophore cultured on Jiffy-7. White arrows show bacterial clump (blackish one).
